# Supplementary material for: Metagenomic Insights for Antimicrobial Resistance Surveillance in Soils with Different Land Uses in Brazil
Source: Antibiotics (Basel). 2023 Feb 5;12(2):334. doi: 10.3390/antibiotics12020334 (PMC9952835; doi:10.3390/antibiotics12020334)
Supplement: Supplementary file 1 [file antibiotics-12-00334-s001.zip › antibiotics-2113485-supplementary.pdf]

# Metagenomic insights for antimicrobial resistance surveillance in soils with different land uses in Brazil

João Vitor Wagner Ordine[1$], Gabrielle Messias de Souza[1$], Gustavo Tamasco[2], Stela Virgilio[2], Ana Flávia Tonelli Fernandes[1], Rafael Silva-Rocha[2] and María-Eugenia Guazzaroni[1*]

[1] Department of Biology, Faculdade de Filosofia, Ciências e Letras de Ribeirão Preto, University of São Paulo, Ribeirão Preto, SP, Brazil

[2] ByMyCell Inova Simples. Av. Dra. Nadir Águiar, 1805 – Supera Parque, Ribeirão Preto, SP, Brazil

[$]Both authors contributed equally to this work

*Correspondence to: María-Eugenia Guazzaroni, meguazzaroni@ffclrp.usp.br
Faculdade de Filosofia, Ciências e Letras de Ribeirão Preto, Universidade de São Paulo.
Av. Bandeirantes, 3.900. CEP: 14049-901, Ribeirão Preto, São Paulo, Brazil. Tel +55 (16) 3315 3680

## Supporting Materials

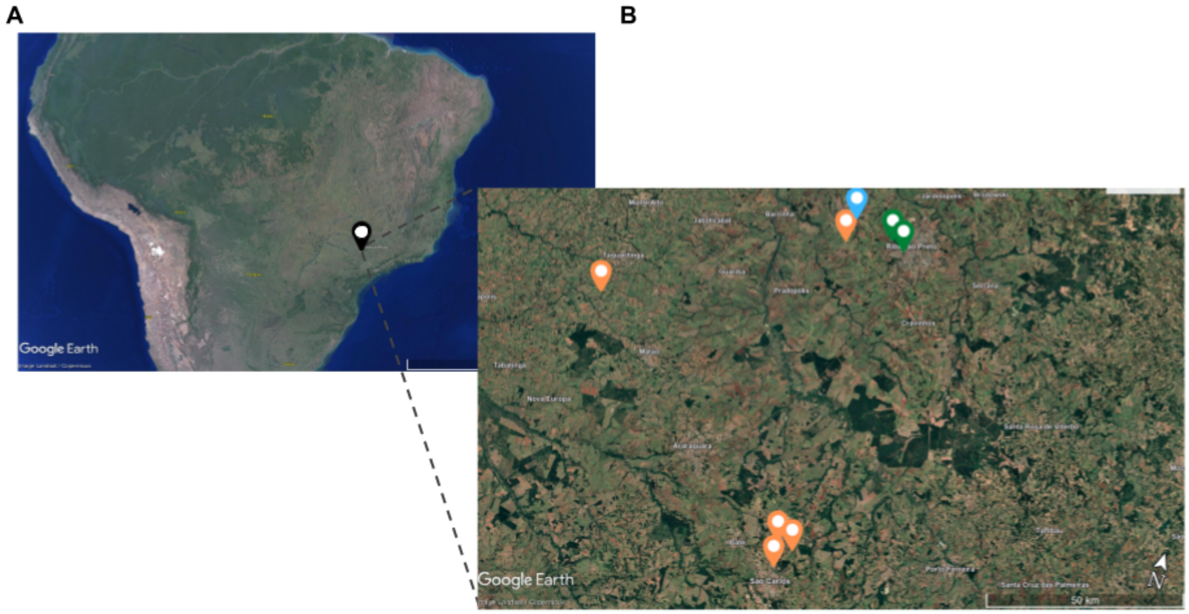


**Figure S1.** Map of soil sample locations. **(A)** Brazil map, with Ribeirão Preto marked in green. **(B)** Part of the São Paulo northeastern region, in which collection sites, along with their land use classification, are shown with green (forest), blue (urban) and orange (farming soils) markers. The map was taken using Google Maps and Google Earth Pro (https://www.google.cl/maps; accessed on 29 July 2022).

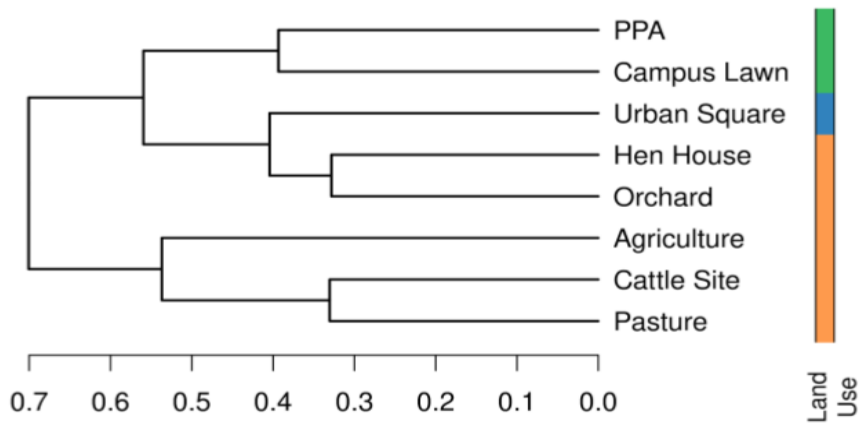


**Figure S2.** Soil sample clusterization based on microbial composition. The cladogram was constructed using Ward's algorithm for hierarchical clustering on a distance matrix obtained with Bray-Curtis dissimilarity method.

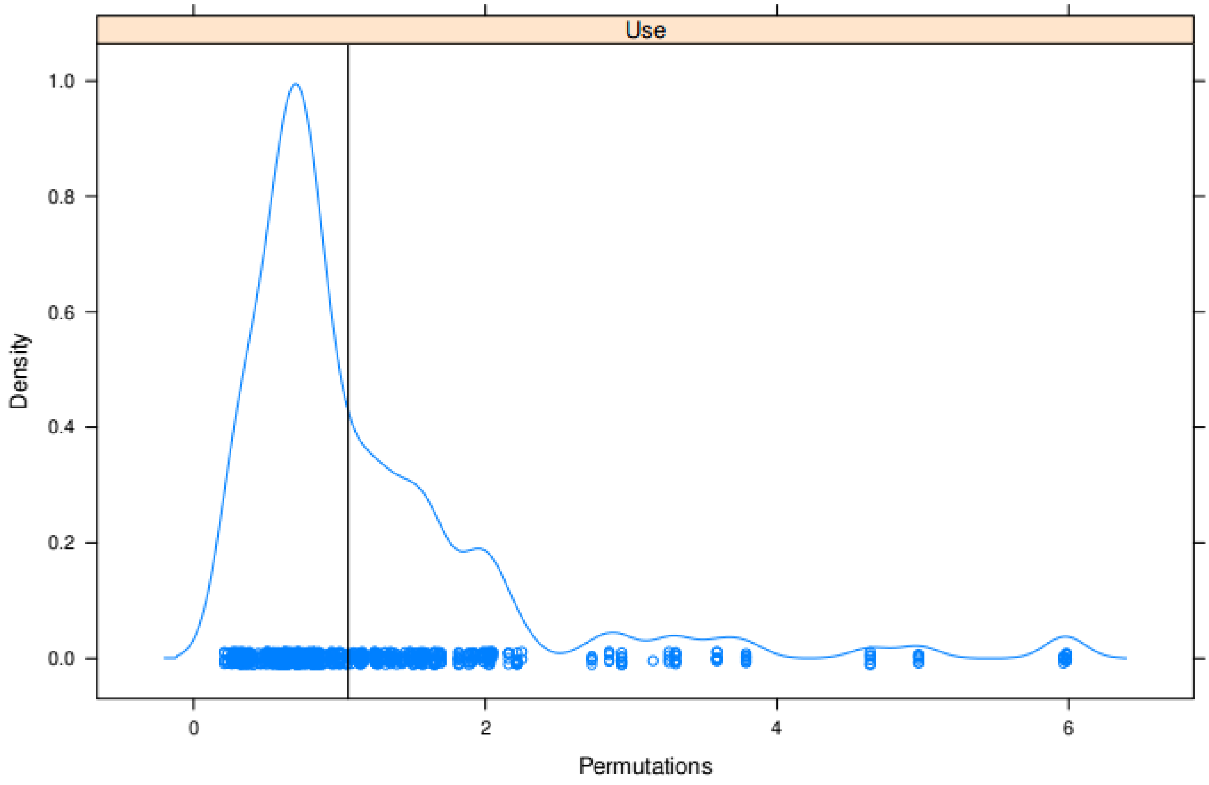


**Figure S3.** Permutation of the resistome profiles. Data was shuffled 1000 times to test the null hypothesis (data is not different from randomly shuffled data). Permuted F-values are represented by the blue distribution line, the F-value of the study dataset is represented by the black line.

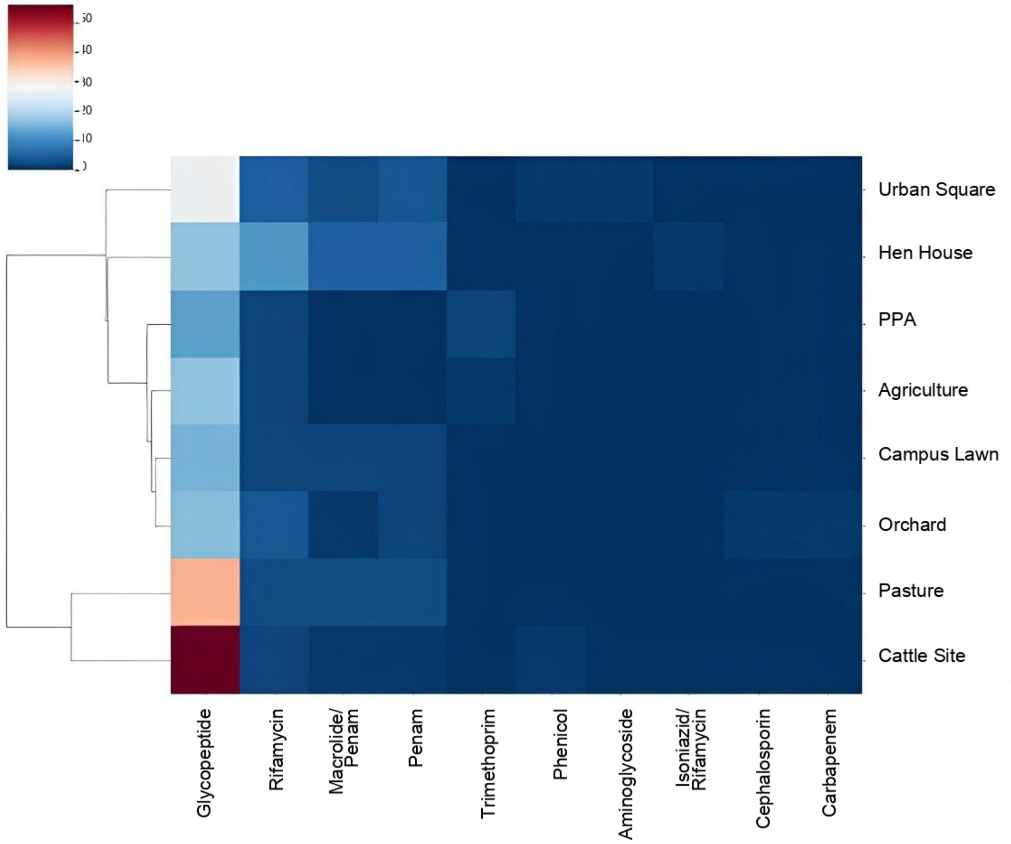


**Figure S4.** Heatmap showing the presence of ARGs identified by CARD indicating the number of each resistance gene per soil. Data were clustered using hierarchical mapping with Euclidean distance. The blue to red scale indicates the number of ARGs for each soil sample in each category.

**Table S1.** Shotgun sequencing - Illumina NovaSeq 6000- data quality summary.

| Sample ID* | Raw Reads | Raw data (G) | Effective (%) | Error (%) | Q20 (%) | Q30 (%) | GC (%) |
|---|---|---|---|---|---|---|---|
| SOrch | 117562592 | 17.6 | 99.87 | 0.03 | 97.36 | 92.70 | 62.79 |
| SLawn | 95499316 | 14.3 | 99.84 | 0.03 | 97.73 | 93.48 | 61.67 |
| SPas | 107762684 | 16.2 | 99.86 | 0.03 | 97.53 | 93.22 | 66.09 |
| SUrbSq | 120088348 | 18.0 | 99.84 | 0.03 | 97.57 | 93.23 | 65.32 |
| SCatt | 124484298 | 18.7 | 99.84 | 0.03 | 97.58 | 93.22 | 66.34 |
| SHen | 104840520 | 15.7 | 99.82 | 0.03 | 97.44 | 92.89 | 65.26 |
| SPpa | 83080300 | 12.5 | 99.82 | 0.02 | 98.04 | 94.51 | 63.29 |
| SAgri | 81025724 | 12.2 | 99.80 | 0.03 | 97.90 | 94.13 | 64.14 |

* SOrch: Orchard; SLawn: Campus Lawn; SPas: Pasture; SUrbSq: Urban Square; SCatt: Cattle Site; SHen: Hen House; SPpa: Permanent Protected Area; SAgri: Agriculture.

**Table S2.** Relative abundances of most abundant genera (above 2.5%) identified in sequenced soils.

| Genus | Soil Sample | Read Count | Relative Abundance |
|---|---|---|---|
| *Occallatibacter* | PPA | 1667 | 0.056 |
| *Pseudolabrys* | PPA | 1483 | 0.050 |
| *Rhodoplanes* | PPA | 1302 | 0.044 |
| *Edaphobacter* | PPA | 1224 | 0.041 |
| *Tumebacillus* | PPA | 1152 | 0.039 |
| *Terrimonas* | PPA | 1083 | 0.037 |
| *Paraburkholderia* | PPA | 890 | 0.030 |
| *Bradyrhizobium* | PPA | 888 | 0.030 |
| *Terriglobus* | PPA | 881 | 0.030 |
| *Vicinamibacter* | PPA | 848 | 0.029 |
| *Lysobacter* | PPA | 764 | 0.026 |
| *Terrimonas* | Campus Lawn | 758 | 0.122 |
| *Lysobacter* | Campus Lawn | 406 | 0.066 |
| *Pseudolabrys* | Campus Lawn | 274 | 0.044 |
| *Occallatibacter* | Campus Lawn | 259 | 0.042 |
| *Haliangium* | Campus Lawn | 229 | 0.037 |
| *Gemmatimonas* | Campus Lawn | 216 | 0.035 |
| *Rhodoplanes* | Campus Lawn | 212 | 0.034 |
| *Vicinamibacter* | Campus Lawn | 207 | 0.033 |
| *Chitinophaga* | Campus Lawn | 192 | 0.031 |
| *Flavisolibacter* | Urban Square | 1684 | 0.048 |
| *Massilia* | Urban Square | 1260 | 0.036 |
| *Gemmatimonas* | Urban Square | 1157 | 0.033 |
| *Haliangium* | Urban Square | 1154 | 0.033 |
| *Vicinamibacter* | Urban Square | 1021 | 0.029 |
| *Rhodoplanes* | Urban Square | 914 | 0.026 |
| *Bacillus* | Agriculture | 335 | 0.312 |
| *Staphylococcus* | Agriculture | 62 | 0.058 |
| *Rhodoplanes* | Agriculture | 33 | 0.031 |
| *Vicinamibacter* | Agriculture | 29 | 0.027 |
| *Tumebacillus* | Agriculture | 28 | 0.026 |
| *Bacillus* | Hen House | 1833 | 0.044 |

| | | | |
|---|---|---|---|
| *Massilia* | Hen House | 1808 | 0.044 |
| *Vicinamibacter* | Hen House | 1352 | 0.033 |
| *Fimbriiglobus* | Hen House | 1260 | 0.030 |
| *Occallatibacter* | Hen House | 1074 | 0.026 |
| *Bacillus* | Pasture | 7529 | 0.177 |
| *Vicinamibacter* | Pasture | 2643 | 0.062 |
| *Rhodoplanes* | Pasture | 1618 | 0.038 |
| *Fimbriiglobus* | Pasture | 1288 | 0.030 |
| *Gaiella* | Pasture | 1119 | 0.026 |
| *Bacillus* | Cattle Site | 3017 | 0.118 |
| *Vicinamibacter* | Cattle Site | 1304 | 0.051 |
| *Gaiella* | Cattle Site | 907 | 0.035 |
| *Rhodoplanes* | Cattle Site | 805 | 0.031 |
| *Occallatibacter* | Cattle Site | 701 | 0.027 |
| *Paludibaculum* | Cattle Site | 653 | 0.025 |
| *Fimbriiglobus* | Cattle Site | 648 | 0.025 |
| *Fimbriiglobus* | Orchard | 996 | 0.051 |
| *Occallatibacter* | Orchard | 949 | 0.048 |
| *Rhodoplanes* | Orchard | 666 | 0.034 |
| *Bryobacter* | Orchard | 614 | 0.031 |
| *Paludibaculum* | Orchard | 612 | 0.031 |
| *Flavisolibacter* | Orchard | 553 | 0.028 |
| *Terrimonas* | Orchard | 522 | 0.027 |
| *Terriglobus* | Orchard | 520 | 0.026 |
| *Edaphobacter* | Orchard | 503 | 0.026 |

**Table S3.** Alpha diversity indexes of sampled soils.

| Soil | Shannon Index | Simpson Index |
|---|---|---|
| Hen House | 5.087 | 0.9867 |
| Urban Square | 5.056 | 0.9866 |
| Orchard | 4.859 | 0.9835 |
| Cattle Site | 4.791 | 0.9746 |
| Pasture | 4.543 | 0.9570 |
| PPA | 4.504 | 0.9783 |
| Campus Lawn | 4.343 | 0.9670 |
| Agriculture | 3.781 | 0.8932 |

**Table S4.** Data availability. The trimmed sequences, along with their quality scores, resulting from shotgun sequencing are publicly available under the BioProject PRJNA900430.

| BioSample Accession | Sample Name/SPUID | Organism | Tax ID | BioProject | SRA Accession |
|---|---|---|---|---|---|
| SAMN31691838 | SHen | soil metagenome | 410658 | PRJNA900430 | SRR22278232 |
| SAMN31691839 | SCatt | soil metagenome | 410658 | PRJNA900430 | SRR22278231 |
| SAMN31691840 | SLawn | soil metagenome | 410658 | PRJNA900430 | SRR22278230 |
| SAMN31691841 | SUrbSq | soil metagenome | 410658 | PRJNA900430 | SRR22278229 |
| SAMN31691842 | SPast | soil metagenome | 410658 | PRJNA900430 | SRR22278228 |
| SAMN31691843 | SOrch | soil metagenome | 410658 | PRJNA900430 | SRR22278227 |
| SAMN31691844 | SAgri | soil metagenome | 410658 | PRJNA900430 | SRR22278226 |
| SAMN31691845 | SPpa | soil metagenome | 410658 | PRJNA900430 | SRR22278225 |

**Table S5.** Summary table of the identified ARGs by CARD in sequenced metagenomes of soils under different land use systems.

| ARG | Count | Resistance | Soil | Land Use |
|---|---|---|---|---|
| *vanRO* | 15 | Glycopeptide | Campus Lawn | Forest |
| *rbpA* | 2 | Rifamycin | Campus Lawn | Forest |
| *mtrA* | 2 | Macrolide;Penam | Campus Lawn | Forest |
| *vanRO* | 13 | Glycopeptide | PPA | Forest |
| *dfrB7_1* | 1 | Trimethoprim | PPA | Forest |
| *dfrB3* | 1 | Trimethoprim | PPA | Forest |
| *rbpA* | 2 | Rifamycin | PPA | Forest |
| **Total Forest** | 36 | | | |
| *cpt* | 1 | Phenicol | Urban Square | Urban |
| *aac2-Ib* | 1 | Aminoglycoside | Urban Square | Urban |
| *bla*F | 1 | Beta-lactamase | Urban Square | Urban |
| *vanRO* | 26 | Glycopeptide | Urban Square | Urban |
| *rbpA* | 5 | Rifamycin | Urban Square | Urban |
| *mtrA* | 3 | Macrolide;Penam | Urban Square | Urban |
| **Total Urban** | 37 | | | |
| *mtrA* | 5 | Macrolide;Penam | Hen House | Farming |
| *rbpA* | 11 | Rifamycin | Hen House | Farming |
| *vanRO* | 17 | Glycopeptide | Hen House | Farming |
| *efpA* | 1 | Isoniazid;Rifamycin | Hen House | Farming |
| Total | 34 | | | |
| *blaLRA-9* | 1 | Beta-lactamase | Orchard | Farming |
| *blaBJP-1* | 1 | Beta-lactamase | Orchard | Farming |
| *vanRO* | 15 | Glycopeptide | Orchard | Farming |
| *rbpA* | 4 | Rifamycin | Orchard | Farming |
| *mtrA* | 1 | Macrolide;Penam | Orchard | Farming |
| *vanSO* | 1 | Glycopeptide | Orchard | Farming |
| Total | 23 | | | |
| *vanRO* | 37 | Glycopeptide | Pasture | Farming |

| | | | | |
|---|---|---|---|---|
| *rbpA* | 3 | Rifamycin | Pasture | Farming |
| *vanSO* | 1 | Glycopeptide | Pasture | Farming |
| *mtrA* | 3 | Macrolide;Penam | Pasture | Farming |
| Total | 44 | | | |
| *cpt* | 1 | Phenicol | Cattle Site | Farming |
| *vanRO* | 54 | Glycopeptide | Cattle Site | Farming |
| *rbpA* | 2 | Rifamycin | Cattle Site | Farming |
| *vanSO* | 2 | Glycopeptide | Cattle Site | Farming |
| *mtrA* | 1 | Macrolide;Penam | Cattle Site | Farming |
| Total | 60 | | | |
| *dfrB3* | 1 | Trimethoprim | Agriculture | Farming |
| *rbpA* | 2 | Rifamycin | Agriculture | Farming |
| *vanRO* | 17 | Glycopeptide | Agriculture | Farming |
| Total | 20 | | | |
| **Total Farming** | 181 | | | |

**Table S6.** Summary table of the identified VFs by VFDB in sequenced metagenomes of soils under different land use systems.

| VF | Count | Soil | Land Use |
|---|---|---|---|
| *acpXL* | 8 | Campus Lawn | Forest |
| *pilG* | 1 | Campus Lawn | Forest |
| *hsiB1/vipA* | 1 | Campus Lawn | Forest |
| *icl* | 1 | Campus Lawn | Forest |
| **Total** | **11** | | |
| *acpXL* | 6 | PPA | Forest |
| *hsiB1/vipA* | 4 | PPA | Forest |
| *pilG* | 3 | PPA | Forest |
| **Total** | **13** | | |

**Table S7.** Summary table of the identified VFs by VFDB in sequenced soil metagenomes on urban land use system.

| VF | Count | Soil | Land Use |
|---|---|---|---|
| *algW* | 1 | Urban Square | Urban |
| *flgC* | 2 | Urban Square | Urban |
| *esxH* | 1 | Urban Square | Urban |
| *fliE* | 1 | Urban Square | Urban |
| *acpXL* | 9 | Urban Square | Urban |
| *hsiB1/vipA* | 1 | Urban Square | Urban |
| *esxN* | 2 | Urban Square | Urban |
| *waaG* | 1 | Urban Square | Urban |
| *mucD* | 1 | Urban Square | Urban |
| *pilT* | 1 | Urban Square | Urban |
| *pilM* | 1 | Urban Square | Urban |
| *fliQ* | 1 | Urban Square | Urban |
| *fliP* | 1 | Urban Square | Urban |
| *fliN* | 1 | Urban Square | Urban |
| *esxM* | 2 | Urban Square | Urban |
| *phoP* | 1 | Urban Square | Urban |
| *icl* | 1 | Urban Square | Urban |
| *pilG* | 1 | Urban Square | Urban |
| *pilH* | 1 | Urban Square | Urban |
| *algR* | 1 | Urban Square | Urban |
| *fliA* | 1 | Urban Square | Urban |
| **Total** | **32** | | |

**Table S8.** Summary table of the identified VFs by VFDB in sequenced soil metagenomes on farming land use system.

| VF | Count | Soil | Land Use |
|---|---|---|---|
| *phoP* | 1 | Hen House | Farming |
| *acpXL* | 10 | Hen House | Farming |
| *cheW* | 1 | Hen House | Farming |
| *esxH* | 1 | Hen House | Farming |
| *hsiB1/vipA* | 2 | Hen House | Farming |
| *mbtH* | 2 | Hen House | Farming |
| *ideR* | 1 | Hen House | Farming |
| *pilG* | 2 | Hen House | Farming |
| *icl* | 1 | Hen House | Farming |
| **Total** | **21** | | |
| *phoP* | 2 | Orchard | Farming |
| *mbtH* | 2 | Orchard | Farming |
| *pilT* | 1 | Orchard | Farming |
| *hsiB1/vipA* | 4 | Orchard | Farming |
| *esxM* | 4 | Orchard | Farming |
| *acpXL* | 10 | Orchard | Farming |
| *esxN* | 4 | Orchard | Farming |
| *ideR* | 1 | Orchard | Farming |
| *pilG* | 1 | Orchard | Farming |
| **TOTAL** | **29** | | |
| *acpXL* | 9 | Pasture | Farming |
| *mbtH* | 1 | Pasture | Farming |
| *hsiB1/vipA* | 3 | Pasture | Farming |
| **TOTAL** | **13** | | |
| *pilG* | 1 | Cattle Site | Farming |
| *acpXL* | 8 | Cattle Site | Farming |
| **TOTAL** | **9** | | |
| *acpXL* | 5 | Agriculture | Farming |
| *pilG* | 2 | Agriculture | Farming |
| *esxM* | 1 | Agriculture | Farming |
| *icl* | 1 | Agriculture | Farming |
| *pilT* | 2 | Agriculture | Farming |
| *fliA* | 1 | Agriculture | Farming |
| *esxG* | 1 | Agriculture | Farming |
| *hsiB1/vipA* | 1 | Agriculture | Farming |
| *phoP* | 1 | Agriculture | Farming |
| **Total** | **15** | | |
| **Total Farming** | **143** | | |
